# The Use of Erector Spinae Plane Block Reduces Opioid Consumption and Pain Score in Postoperative Period After Hip Surgery: A Meta-Analysis

**DOI:** 10.7759/cureus.47477

**Published:** 2023-10-22

**Authors:** Anwar U Huda, Hashsaam Ghafoor

**Affiliations:** 1 Anesthesiology, Hamad Medical Corporation, Doha, QAT

**Keywords:** vomiting, pain, nausea, hip surgery, espb

## Abstract

Erector spinae plane block (ESPB) is a relatively new regional anesthesia block that has been used in thoracic and abdominal surgeries with variable success. ESPB can easily be administered using an ultrasound technique with a safer profile. Recently, there have been few randomized controlled trials (RCTs) regarding the role of ESPB in hip surgeries. A current meta-analysis was done to evaluate the role of ESPB block in controlling postoperative pain after hip surgeries.

PRISMA guidelines were followed to perform this meta-analysis. We used online databases including Science Direct, PubMed, Google Scholar, and Cochrane Library. This review was registered with the International Prospective Register of Systematic Reviews (PROSPERO) database as ID-CRD42023445516 in July 2023. We included studies that reported opioid use, pain control after surgery, and side effects associated with ESPB for hip surgeries. The ReviewManager software, i.e., RevMan for Mac 5.4 (Cochrane Collaboration, Oxford, UK), was utilized to conduct this meta-analysis.

We included five RCTs during this meta-analysis. Our results demonstrated that the use of ESPB in hip surgery caused a significant decrease in 24-hour postoperative opioid consumption (p=0.02). ESPB also resulted in a significant decrease in pain scores up to nine hours postoperatively (p<0.05).

## Introduction and background

Hip surgeries have become one of the more frequently performed procedures over the last 20 years, likely due to higher life expectancy and also altered lifestyles culminating in the obesity epidemic. Inadequate pain control postoperatively can result in delays in ambulation and physiotherapy and, hence, poor functional recovery and patient satisfaction. Additionally, it can prolong hospital stays and increase the risk of complications like deep vein thrombosis [1,2].

Because of the complex nature of the sensory supply of the hip joint, complete analgesia for hip surgery is obtained by blocking the femoral nerve, sciatic nerve, obturator nerve, superior gluteal nerve, and the nerve to quadratus femoris [3]. Blockage of these nerves separately is cumbersome and carries more risk of nerve injuries. Various single-shot injection techniques have been used to provide adequate pain relief after hip surgeries, which include fascia iliaca block, femoral nerve block, lumbar plexus block, pericapsular nerve group block, and quadratus lumborum block [4]. Most of these techniques carry the potential risk of motor weakness of quadriceps muscles which can delay early mobilization [3].

Erector spinae plane block (ESPB) is a novel regional block that has the advantage of blocking both visceral and somatic nerve fibers. Additionally, it can be performed using easily identified ultrasound landmarks with a much better safety profile [5,6]. It has been used with variable success in breast surgery [7], thoracic surgery, bariatric surgery, and nephrectomy [8,9]. There have been a few studies relevant to the use of ESPB for hip surgeries [10-14]. We conducted this meta-analysis to assess the efficacy of ESPB on postoperative pain control and opioid consumption after hip surgeries compared with no block.

## Review

Methods

This systematic review and meta-analysis was conducted in July-September 2023. The initial search was done in July 2023 followed by another in August 2023 to ensure accuracy. The review was registered with the International Prospective Register of Systematic Reviews (PROSPERO) as ID-CDR CRD42023445516 in July 2023. Our search and review were performed in accordance with the Preferred Reporting Items for Systematic Reviews and Meta-Analyses (PRISMA) guidelines [15].

We developed a search strategy for Science Direct, PubMed, Google Scholar, and Cochrane Library, utilizing "erector spinae plane block" OR "ESPB" AND "hip arthroplasty" OR "hip surgery." It consisted of randomized controlled trials (RCTs) published in English and related to patients who received ESPB for hip surgeries. The primary outcome was 24-hour opioid consumption in the postoperative period. The secondary outcomes were postoperative pain scores and the occurrence of adverse events including postoperative nausea and vomiting (PONV). Only primary research was considered for our review, while we excluded abstracts, comments, technique articles, and review articles.

The data was extracted from all included studies using a pre-designed proforma regarding populations and outcomes. We included information like the studies' general details (name of journal, year of publication, design, groups, and outcomes), study participants, sample size, intervention (doses and administration timings), and outcomes (24-hour opioid consumption, pain control, and side effects). Means and standard deviations of continuous variables were retrieved from the tables or graphs.

The two researchers performed the search independently. All discrepancies were resolved through constructive arguments between the researchers. The Consolidated Standards of Reporting Trials checklist was utilized to appraise the individual studies by both researchers [16].

We assessed the quality of the included studies by using the risk of bias 2 tool (RoB2; Cochrane, London, United Kingdom) [17]. ReviewManager (RevMan for Mac, version 5.4; Cochrane Collaboration, Oxford, UK) was utilized to perform the meta-analysis of the included studies. The value of I2 was measured to assess the heterogeneity of data. Data on 24-hour opioid consumption was pooled. Similarly, data on postoperative pain control and incidence of adverse events were also pooled. For continuous data, the mean difference or standard mean difference was used to report the treatment effect, while the odds ratio was used for dichotomous data. A random effect model was used due to expected heterogeneity in the included studies. A p-value of less than 0.05 was set as the level of statistical significance.

Results

**Figure 1:**
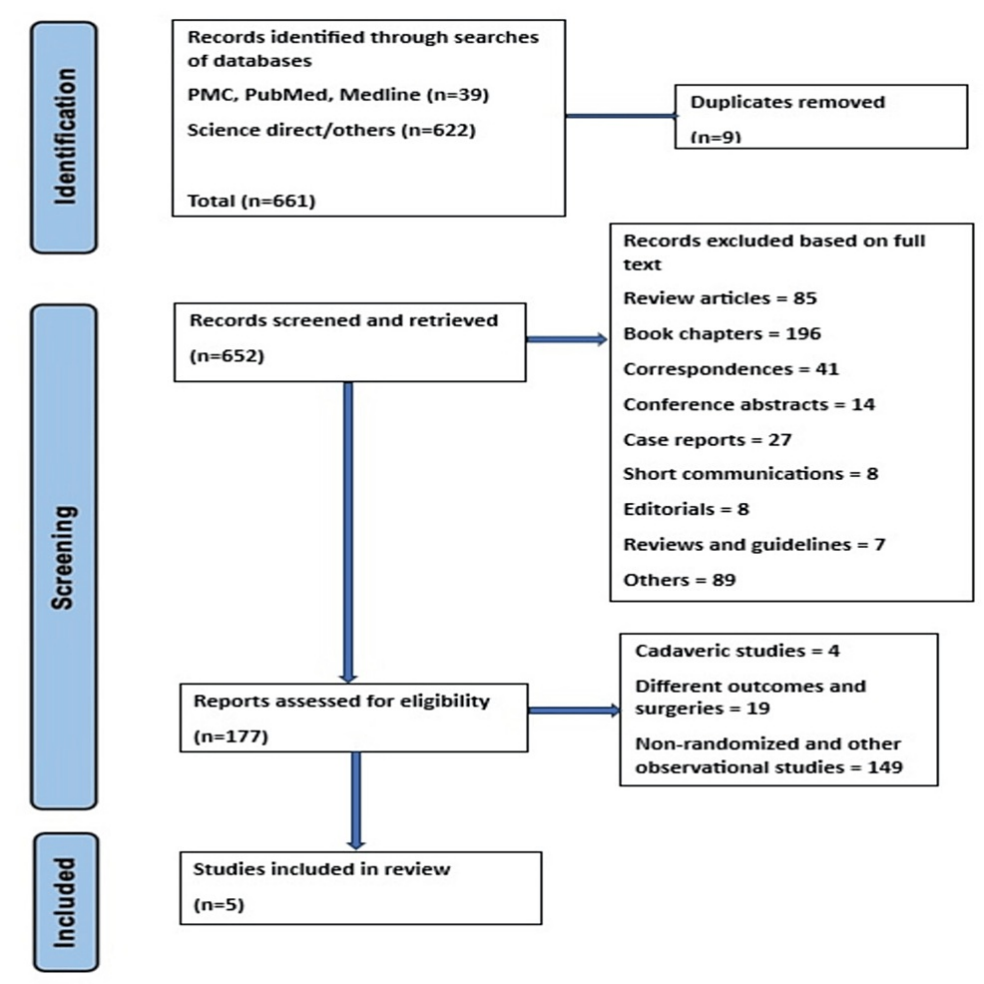
PRISMA flowchart

We found 661 studies by using our search strategy including "erector spinae plane block" OR "ESPB" AND "hip surgery" OR "hip arthroplasty" in Science Direct, PubMed, Google Scholar, and Cochrane Library. After a thorough assessment of studies as demonstrated in Figure 1, we identified five studies to be included in our systematic review.

A brief detail of all included studies [10,18-21] is illustrated in Table 1.

**Table 1:** Concise details of included studies ASA1: American Society of Anesthesiologists Classification 1, THR: total hip replacement, FAIS: femoroacetabular impingement syndrome, L-ESB: lumbar erector spinae plane block, QLB-t: quadratus lumborum block, ESP: erector spinae plane, PONV: postoperative nausea and vomiting, QoR: quality of recovery

Study	Population	Intervention (s)	Comparator	Outcome	Results
Tulgar et al., 2018 [10] N=60	ASA1 to III patients undergoing hip and femur surgeries	L-ESB, QLB-t	Control group	The primary outcome was pain scores at 0-24 hours postoperatively	Pain scores were different at the first, third, and sixth hours postoperatively. Lower opioid consumption in the intervention group at 12 and 24 hours. Higher number of patients in the control group needed rescue analgesia
Chan et al., 2022 [18] N= 71	ASA I to III primary elective unilateral THR	40 mL of 0.25% levobupivacaine injected into the ESP	No block. Both groups received IV fentanyl infusion at 4 mcg/kg in the first hour followed by 2 and then 1 mcg/kg/hr intraoperatively	The primary outcomes were opioid consumption and pain scores in the first 24 hours. The secondary outcome was the incidence of adverse events	Opioid consumption was not significantly different. Pain scores were similar in both groups. Higher incidence of vomiting in the control group
Townsend et al., 2022 [19] N=63	ASA I to III elective primary total hip arthroplasty	Lumbar ESPB with 30 ml of 0.375% ropivacaine	Control group (no block)	The primary outcome was total opioid consumption at 24 hours postoperatively The secondary outcomes were opioid consumption at 8 and 48 hours postoperatively and pain scores at 24 and 48 hours postoperatively	Higher opioid consumption in the first 8 hours in the control group. No significant difference in opioid consumption at 24 and 48 hours postoperatively. No significant difference in pain scores between the two groups
Zimmerer et al., 2022 [20] N=68	Patient age more than 18 years having FAIS	ESPB with 30 ml of 0.375% ropivacaine	Sham block preoperatively with 30 mL of 0.9% saline	The primary outcomes were pain scores during 24 hours postoperatively The secondary outcomes were 24 hours of opioid consumption and incidence of PONV	Pain scores were significantly lower during the first 24 hours in the ESPB group. No significant difference in opioid consumption and incidence of postoperative nausea
Lennon et al., 2020 [21] N=64	Patients scheduled for elective primary unilateral hip arthroplasty	ESPB at the third lumbar vertebra with 30 ml of 0.2% ropivacaine	30 ml of 0.9% saline was used (control group)	The primary outcome was pain score on movement at 6 hours postoperatively The secondary outcomes were quality of recovery (QoR-15 score), mobilization, and length of stay	No significant difference in pain scores at any time. No significant difference in the quality of recovery between the two groups. Length of stay was insignificantly different between the two groups

RoB2 bias assessment tool assesses all included studies to have a low risk of bias.

**Figure 2:**
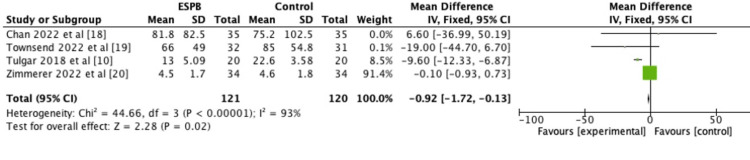
Forest plot of 24-hour opioid consumption p-value equal to or less than 0.05 is considered significant ESPB: erector spinae plane block

The pooled meta-analysis of the included studies [10,18-20] showed that 24-hour opioid consumption was significantly lesser in the group that received ESPB compared to no block (p=0.02) as demonstrated in Figure 2.

**Figure 3:**
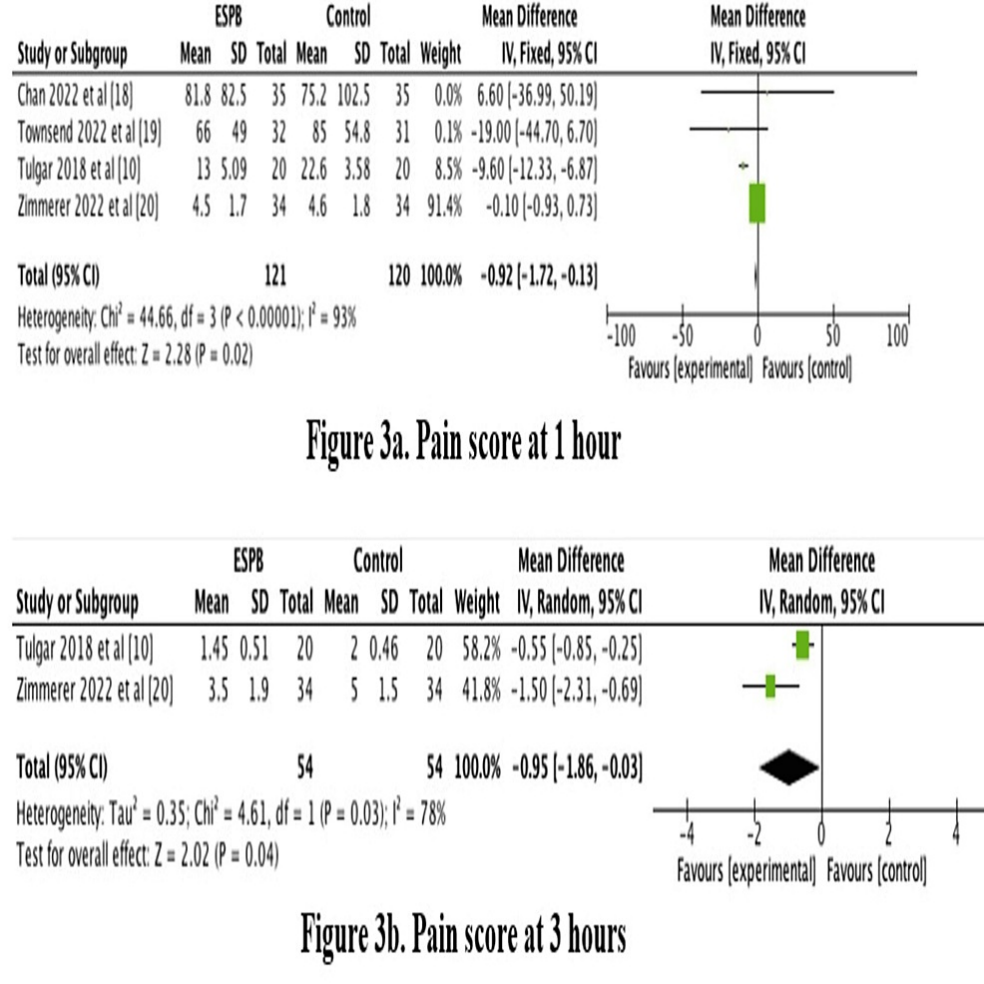
Forest plot showing pain scores postoperatively p-value equal to or less than 0.05 is considered significant

**Figure 4:**
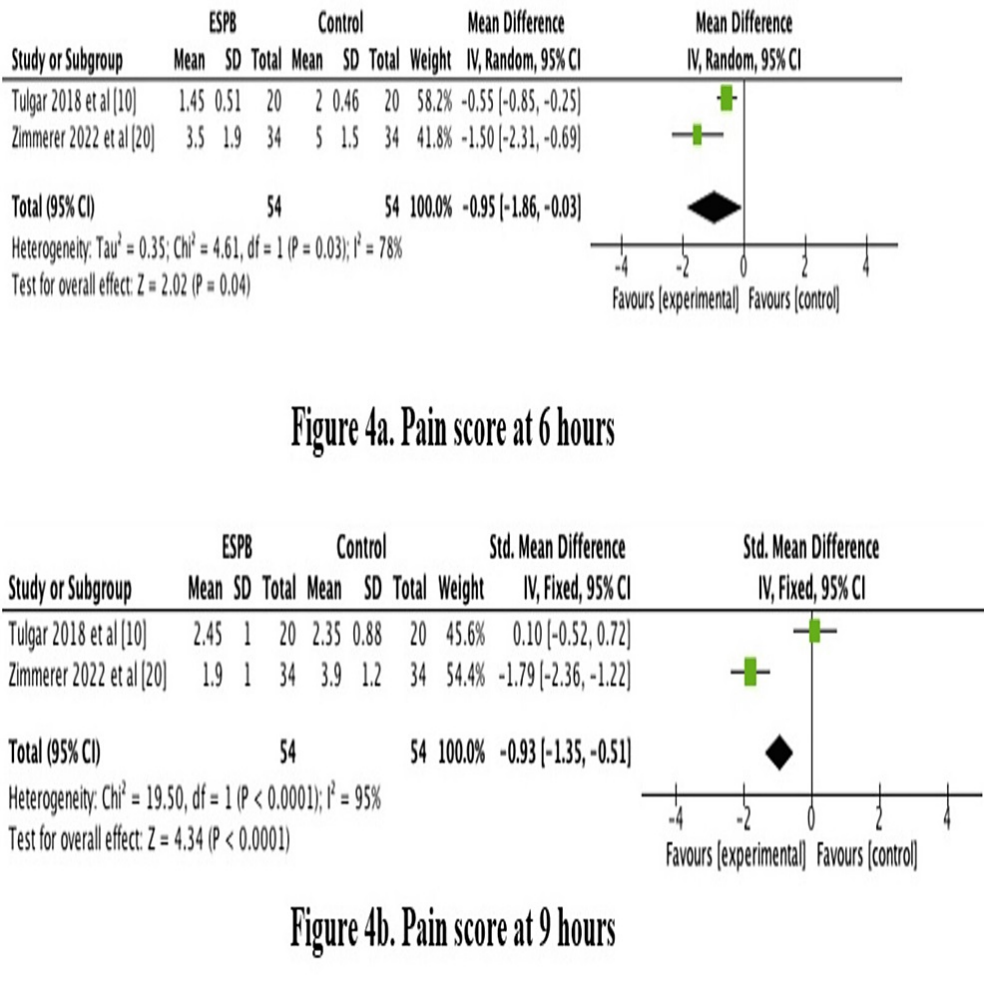
Forest plot showing pain scores postoperatively p-value equal to or less than 0.05 is considered significant

Pain scores were significantly lower in the ESPB group at one, three, six, and nine hours postoperatively with p-values of 0.0007, 0.04, 0.002, and <0.0001, respectively, as shown in Figure 3. However, pain scores at 12 and 24 hours were not statistically different between the two groups with p-values of 0.20 and 0.54, respectively, as shown in Figures 3-4.

The incidence of PONV was lower in the ESPB group compared to the control group (6 vs. 14), although it did not reach statistical significance (p=0.06).

Discussion

Our meta-analysis demonstrated that the use of ESPB in hip surgeries decreased 24-hour opioid consumption. It also reduced numerical rating scale pain scores in the first nine hours postoperatively. There was no associated increased risk of complications.

Our understanding of the exact mechanism of action of ESPB is limited. The local anesthetic solution injected in ESP may reach anteriorly to the epidural, paravertebral, and intervertebral foramen, although this spread could be prevented by the presence of various structures at thoracic and lumbar levels [22,23]. The main structures at the lumbar level that limit the spread anteriorly include the anterior and middle thoracolumbar fascia, intertransverse ligaments, and muscles [24]. There exist frequent discrepancies in the findings from cadaver vs. living human studies regarding the spread of injectate in ESPB. Two cadaver studies demonstrated that injectate only spread to the dorsal rami and not to lumbar plexus roots [25,26]. However, Tulgar et al. [27] showed that lumbar ESPB for hip surgery resulted in the spread of injected dye at the L3-5 roots in the psoas compartment. Despite the discrepancy, ESPB is considered a regional anesthetic block for hip surgeries due to its safe approach and its multi-level spread of local anesthetic by a single injection [11,12].

Our results demonstrated a significant decrease in 24-hour opioid consumption after ESPB in hip surgeries, which was consistent with the findings in another meta-analysis done with ESPB in breast cancer surgeries [28]. In our meta-analysis, the mean 24-hour opioid consumption was 43.59 and 58.99 mg in the ESPB and control group, respectively, and the standard mean difference was -0.30. Liu et al. [29] reported a significant reduction in 24-hour opioid consumption in the ESPB group done for lumbar spine surgery with an SMD = -2.80, p<0.0001. Ma et al. [30] also reported a significant reduction in 24-hour opioid consumption by using ESPB in spine surgery with an SMD = -1.834, p<0.001. Our meta-analysis found a mean difference of -15.4 mg with the use of ESPB in hip surgeries. Leong et al. [31] showed that ESPB significantly reduced opioid requirement in 24 hours postoperatively after breast surgery with a mean difference of -21.55 (-32.57, -10.52) and p=0.001. Another meta-analysis done by Huang et al. [32] for breast and thoracic surgery demonstrated a significant reduction in 24-hour opioid consumption with a relatively lower mean difference of -10.5 mg compared to our results.

Our results demonstrated a significant decrease in pain scores up to nine hours postoperatively with the use of ESPB for hip surgeries. Liu et al. [29] reported a significant decrease in pain scores at 2, 6, 12, and 24 hours postoperatively in the meta-analysis done in patients undergoing lumbar spine surgery. Ma et al. [30] also showed a significant reduction in pain scores up to 24 hours postoperatively by using ESPB for spine surgery. In another meta-analysis of patients undergoing breast surgery, Leong et al. [31] demonstrated a significant decrease in pain scores from 0-24 hours postoperatively by using ESPB. On the other hand, Ribeiro et al. [33] showed that pain scores were insignificantly different between ESPB and control groups at all time frames postoperatively in patients who underwent cesarean section.

In our meta-analysis, the incidence of PONV was found to be lower in the ESPB group compared to the control group (6 vs. 14 respectively), although the difference did not reach statistical significance. This was consistent with the findings in another meta-analysis by Daghmouri et al. [34]. This meta-analysis was done in patients undergoing laparoscopic cholecystectomy. They showed that there was no significant difference in the incidence of nausea between the two groups (OR=0.46, p=0.20). In addition, they did not find any significant difference in the incidence of vomiting (OR= 0.37, p=0.13). Ma et al. [30] on the other hand found a significant difference in PONV by using ESPB in patients undergoing spine surgery. Huang et al. [32] and Sun et al. [35] also reported a significant decrease in PONV with the use of ESPB (p=0.01, p<0.001 respectively).

There were a few limitations in our study. Firstly, three out of five studies were done on patients undergoing total hip arthroplasty, while Tulgar et al. [10] performed a study on patients undergoing hip and proximal femur surgery and Zimmerer et al. [20] on patients undergoing hip arthroscopy. Another limitation of our meta-analysis was that three of the included studies used general anesthesia for their patients, while one study used both general anesthesia and spinal anesthesia and one study used only spinal anesthesia as an anesthetic choice. Among studies using general anesthesia, two studies used ESPB post-induction of anesthesia, while in one study, ESPB was performed pre-induction. Lennon et al. [21] in their study performed spinal anesthesia first followed by ESPB and then general anesthesia. Townsend et al. [19] in their study performed ESPB before doing spinal anesthesia.

The dose and concentration of local anesthetic used for ESPB also varied. Zimmerer et al. [20] and Townsend et al. [19] both used 30 ml of 0.375% ropivacaine for their blocks. Lennon et al. [21] used 30 ml of 0.2% ropivacaine in their blocks. Chan et al. [18] used a higher volume of 40 ml 0.25 % levobupivacaine for ESPB. Tulgar et al. [10] used a solution containing 20 ml 0.5 bupivacaine, 10 ml each of 2% lidocaine, and normal saline in their ESPB.

## Conclusions

Pre-incision use of ESPB can significantly reduce 24-hour opioid consumption after hip surgery. It can also decrease the pain scores up to nine hours postoperatively, although pain scores were not different after nine hours by using ESPB. There was no difference in the incidence of adverse events between the two groups. Further studies comparing its efficacy with other blocks used for hip surgery are recommended.

## References

[1] Wolford ML, Palso K, Bercovitz A (2015). Hospitalization for total hip replacement among inpatients aged 45 and over: United States, 2000-2010. NCHS Data Brief.

[2] Bernstein J, Feng J, Mahure S, Schwarzkopf R, Long W (2020). Revision total knee arthroplasty is associated with significantly higher opioid consumption as compared with primary total knee arthroplasty in the acute postoperative period. Arthroplast Today.

[3] Bugada D, Bellini V, Lorini LF, Mariano ER (2018). Update on selective regional analgesia for hip surgery patients. Anesthesiol Clin.

[4] Huda AU, Ghafoor H (2022). The use of pericapsular nerve group (peng) block in hip surgeries is associated with a reduction in opioid consumption, less motor block, and better patient satisfaction: a meta-analysis. Cureus.

[5] Chin KJ, Malhas L, Perlas A (2017). The erector spinae plane block provides visceral abdominal analgesia in bariatric surgery: a report of 3 cases. Reg Anesth Pain Med.

[6] El-Boghdadly K, Pawa A (2017). The erector spinae plane block: plane and simple. Anaesthesia.

[7] Altıparmak B, Korkmaz Toker M, Uysal Aİ, Gümüş Demirbilek S (2019). Comparison of the efficacy of erector spinae plane block performed with different concentrations of bupivacaine on postoperative analgesia after mastectomy surgery: ramdomized, prospective, double blinded trial. BMC Anesthesiol.

[8] Canturk M (2019). Lumbar erector spinae plane block for postoperative analgesia after nephrectomy followed by emergent complication surgery. Minerva Anestesiol.

[9] Forero M, Rajarathinam M, Adhikary SD, Chin KJ (2018). Erector spinae plane block for the management of chronic shoulder pain: a case report. Can J Anaesth.

[10] Tulgar S, Kose HC, Selvi O, Senturk O, Thomas DT, Ermis MN, Ozer Z (2018). Comparison of ultrasound-guided lumbar erector spinae plane block and transmuscular quadratus lumborum block for postoperative analgesia in hip and proximal femur surgery: a prospective randomized feasibility study. Anesth Essays Res.

[11] Ahiskalioglu A, Tulgar S, Celik M, Ozer Z, Alici HA, Aydin ME (2020). Lumbar erector spinae plane block as a main anesthetic method for hip surgery in high risk elderly patients: initial experience with a magnetic resonance imaging. Eurasian J Med.

[12] Bugada D, Zarcone AG, Manini M, Lorini LF (2019). Continuous erector spinae block at lumbar level (l4) for prolonged postoperative analgesia after hip surgery. J Clin Anesth.

[13] Kinjo S, Schultz A (2019). Continuous lumbar erector spinae plane block for postoperative pain management in revision hip surgery: a case report [Article in Portuguese]. Braz J Anesthesiol.

[14] Singh S, Ranjan R, Lalin D (2019). A new indication of erector spinae plane block for perioperative analgesia is total hip replacement surgery - a case report. Indian J Anaesth.

[15] Shamseer L, Moher D, Clarke M (2015). Preferred reporting items for systematic review and meta-analysis protocols (PRISMA-P) 2015: elaboration and explanation. BMJ.

[16] Falci SG, Marques LS (2015). CONSORT: when and how to use it. Dental Press J Orthod.

[17] Minozzi S, Cinquini M, Gianola S, Gonzalez-Lorenzo M, Banzi R (2020). The revised Cochrane risk of bias tool for randomized trials (RoB 2) showed low interrater reliability and challenges in its application. J Clin Epidemiol.

[18] Chan A, Ng TK, Tang BY (2022). Single-shot lumbar erector spinae plane block in total hip replacement: a randomized clinical trial. Anesth Analg.

[19] Townsend D, Siddique N, Kimura A (2022). Lumbar erector spinae plane block for total hip arthroplasty comparing 24-hour opioid requirements: a randomized controlled study. Anesthesiol Res Pract.

[20] Zimmerer A, Schneider MM, Sobau C, Miehlke W, Eichler F, Wawer Matos J (2022). The erector spinae plane block in the setting of hip arthroscopy: a prospective randomized controlled clinical trial. Arthroscopy.

[21] Lennon MJ, Isaac S, Currigan D, O'Leary S, Khan RJ, Fick DP (2021). Erector spinae plane block combined with local infiltration analgesia for total hip arthroplasty: a randomized, placebo controlled, clinical trial. J Clin Anesth.

[22] Bonvicini D, Boscolo-Berto R, De Cassai A (2021). Anatomical basis of erector spinae plane block: a dissection and histotopographic pilot study. J Anesth.

[23] Chin KJ, Lirk P, Hollmann MW, Schwarz SK (2021). Mechanisms of action of fascial plane blocks: a narrative review. Reg Anesth Pain Med.

[24] Tulgar S, Ahiskalioglu A, Aydin ME, Jadon A, Forero M, Gürkan Y (2021). Lumbar erector spinae plane block: a miracle or self-persuasion?. Reg Anesth Pain Med.

[25] Elsharkawy H, Bajracharya GR, El-Boghdadly K, Drake RL, Mariano ER (2019). Comparing two posterior quadratus lumborum block approaches with low thoracic erector spinae plane block: an anatomic study. Reg Anesth Pain Med.

[26] Ivanusic J, Konishi Y, Barrington MJ (2018). A cadaveric study investigating the mechanism of action of erector spinae blockade. Reg Anesth Pain Med.

[27] Tulgar S, Senturk O (2018). Ultrasound guided erector spinae plane block at L-4 transverse process level provides effective postoperative analgesia for total hip arthroplasty. J Clin Anesth.

[28] Hussain N, Brull R, Noble J, Weaver T, Essandoh M, McCartney CJ, Abdallah FW (2021). Statistically significant but clinically unimportant: a systematic review and meta-analysis of the analgesic benefits of erector spinae plane block following breast cancer surgery. Reg Anesth Pain Med.

[29] Liu H, Zhu J, Wen J, Fu Q (2023). Ultrasound-guided erector spinae plane block for postoperative short-term outcomes in lumbar spine surgery: a meta-analysis and systematic review. Medicine (Baltimore).

[30] Ma J, Bi Y, Zhang Y (2021). Erector spinae plane block for postoperative analgesia in spine surgery: a systematic review and meta-analysis. Eur Spine J.

[31] Leong RW, Tan ES, Wong SN, Tan KH, Liu CW (2021). Efficacy of erector spinae plane block for analgesia in breast surgery: a systematic review and meta-analysis. Anaesthesia.

[32] Huang W, Wang W, Xie W, Chen Z, Liu Y (2020). Erector spinae plane block for postoperative analgesia in breast and thoracic surgery: a systematic review and meta-analysis. J Clin Anesth.

[33] Ribeiro Junior ID, Carvalho VH, Brito LG (2022). Erector spinae plane block for analgesia after cesarean delivery: a systematic review with meta-analysis. Braz J Anesthesiol.

[34] Daghmouri MA, Akremi S, Chaouch MA, Mesbahi M, Amouri N, Jaoua H, Ben Fadhel K (2021). Bilateral erector spinae plane block for postoperative analgesia in laparoscopic cholecystectomy: a systematic review and meta-analysis of randomized controlled trials. Pain Pract.

[35] Sun Q, Zhang C, Liu S, Lv H, Liu W, Pan Z, Song Z (2023). Efficacy of erector spinae plane block for postoperative analgesia lumbar surgery: a systematic review and meta-analysis. BMC Anesthesiol.

